# A Function for a Specific Zinc Metalloprotease of African Trypanosomes

**DOI:** 10.1371/journal.ppat.0030150

**Published:** 2007-10-19

**Authors:** Paul M Grandgenett, Keiko Otsu, Helen R Wilson, Mary E Wilson, John E Donelson

**Affiliations:** 1 Interdepartmental Genetics Program, University of Iowa, Iowa City, Iowa, United States of America; 2 Department of Biochemistry, University of Iowa, Iowa City, Iowa, United States of America; 3 Department of Internal Medicine, University of Iowa, Iowa City, Iowa, United States of America; 4 Department of Microbiology, University of Iowa, Iowa City, Iowa, United States of America; 5 Veterans Affairs Medical Center, Iowa City, Iowa, United States of America; University of Georgia, United States of America

## Abstract

The Trypanosoma brucei genome encodes three groups of zinc metalloproteases, each of which contains ∼30% amino acid identity with the major surface protease (MSP, also called GP63) of *Leishmania*. One of these proteases, TbMSP-B, is encoded by four nearly identical, tandem genes transcribed in both bloodstream and procyclic trypanosomes. Earlier work showed that RNA interference against *TbMSP-B* prevents release of a recombinant variant surface glycoprotein (VSG) from procyclic trypanosomes. Here, we used gene deletions to show that TbMSP-B and a phospholipase C (GPI-PLC) act in concert to remove native VSG during differentiation of bloodstream trypanosomes to procyclic form. When the four tandem *TbMSP-B* genes were deleted from both chromosomal alleles, bloodstream *B* ^−/−^ trypanosomes could still differentiate to procyclic form, but VSG was removed more slowly and in a non-truncated form compared to differentiation of wild-type organisms. Similarly, when both alleles of the single-copy GPI-PLC gene were deleted, bloodstream *PLC* ^−/−^ cells could still differentiate. However, when all the genes for both TbMSP-B and GPI-PLC were deleted from the diploid genome, the bloodstream *B* ^−/−^ *PLC* ^−/−^ trypanosomes did not proliferate in the differentiation medium, and 60% of the VSG remained on the cell surface. Inhibitors of cysteine proteases did not affect this result. These findings demonstrate that removal of 60% of the VSG during differentiation from bloodstream to procyclic form is due to the synergistic activities of GPI-PLC and TbMSP-B.

## Author Summary

African trypanosomes cause sleeping sickness, a fatal disease of humans and livestock in Africa. During their life cycle, these protozoan parasites cycle between the bloodstream of mammals and tsetse flies. Their two main developmental stages are the bloodstream form and the procyclic form in the tsetse fly. Bloodstream trypanosomes thwart their host's immune response by periodically switching their major surface protein, the variant surface glycoprotein (VSG). When bloodstream trypanosomes are ingested by a tsetse fly, they must quickly shed the VSG and replace it with an unrelated invariant protein more suited to their survival as procyclic organisms in the fly midgut. Here, we examine the mechanisms used by trypanosomes to remove the VSG during their differentiation from bloodstream to procyclic form in culture. We deleted the genes for one of the trypanosome's protease enzymes from the trypanosome genome and found that bloodstream trypanosomes could still differentiate to the procyclic form, but VSG removal was diminished. Deleting the genes for a phospholipase enzyme had a similar effect—they could still differentiate but VSG removal was impaired. When the genes for both the protease and the phospholipase were deleted, bloodstream trypanosomes could not differentiate to the procyclic form, they retained about 60% of the VSG on their surface, and they died in the differentiation medium. These results highlight the synergistic roles of these two enzymes in the differentiation process.

## Introduction

Trypanosoma brucei is a protozoan parasite that causes African sleeping sickness. Its life cycle consists of several developmental stages in its tsetse fly vector and mammalian host. In the midgut of the tsetse fly, the parasites proliferate as an extracellular procyclic (PRO) form, which have procyclin as their major surface protein. After further development and migration to the tsetse fly's salivary glands, the infective trypanosomes are transmitted to the mammalian host during a blood meal and differentiate into the bloodstream form (BSF) that is also maintained extracellularly in the host [1]. In a process called antigenic variation, BSF trypanosomes evade the host immune system by sequentially expressing different variant surface glycoproteins (VSGs) on their surface [2]. The T. brucei life cycle is completed when another tsetse fly ingests the parasites with its blood meal.

BSF trypanosomes proliferate as long, slender organisms and convert to non-proliferative, short, stumpy organisms that are pre-adapted for differentiation to the PRO form in the tsetse fly midgut [3,4]. A distinguishing feature of the T. brucei life cycle is the rapid shedding of the glycosylphosphatidylinositol (GPI)-linked VSG coat during differentiation from short, stumpy BSF to PRO form [5]. Several reports examining this differentiation in vitro have identified two forms of VSG present in the culture medium within hours of the initiation of differentiation; one is full-length VSG protein that is cleaved from the cell surface by a GPI-specific phospholipase C (GPI-PLC), and the other is a truncated VSG fragment released by a protease [6]. Recently, it has been shown that GPI-PLC can act on surface molecules during the short, stumpy stage and that shedding of VSG during differentiation is also inhibited by peptidomimetic metalloprotease inhibitors, indicating a zinc metalloprotease is involved [7].

The parasitic protozoa Leishmania spp., which are evolutionarily related to T. brucei, occur as an extracellular form in their sand fly vector and an intracellular form in their mammalian hosts' macrophages. All Leishmania spp. contain a major surface zinc metalloprotease (MSP, also called GP63 or leishmanolysin), which has been studied extensively and shown to (i) provide resistance to complement-mediated lysis before *Leishmania* entry into the macrophage, (ii) participate in attachment and entry into the macrophage, and (iii) support survival in the macrophage after entry [8,9]. We previously reported that the T. brucei genome contains multiple genes encoding homologs of *Leishmania* MSP, which have about 33% overall identity to one another and to *Leishmania* MSP [10]. These T. brucei genes can be grouped into three gene families (*TbMSP-A*, *TbMSP-B*, and *TbMSP-C*) on the basis of their different 3′ untranslated regions (UTRs) and their differential expression during the T. brucei life cycle. BSF cells have mRNAs from all three gene families, whereas PRO cells have detectable mRNA from only *TbMSP-B*. In a previous study, we and the laboratory of Jay Bangs at the University of Wisconsin-Madison used RNA interference (RNAi) to demonstrate that T. brucei major surface protease-B (TbMSP-B) can release a recombinant VSG from a transgenic PRO cell line [10]. It has also been shown that when inhibitors of transcription and translation are present during in vitro differentiation from BSF to PRO cells, VSG shedding is incomplete and much of it remains cell-associated [11]. These results suggest that TbMSP-B might be the protease involved in releasing the VSG during differentiation from short, stumpy BSF to PRO cells.

Here, we demonstrate that TbMSP-B is a surface-localized zinc metalloprotease that is expressed predominantly in differentiating BSF-to-PRO cells and in established PRO cells. When all eight genes in the *TbMSP-B* family were deleted from the diploid BSF genome (B ^−/−^ cells), a delayed release of VSG occurred during BSF-to-PRO differentiation in vitro, but cell proliferation during differentiation was unaffected. Likewise, when the two GPI-PLC genes were deleted from the diploid BSF genome (PLC ^−/−^ cells), delayed VSG release during differentiation was also observed and cell proliferation rate was unaffected. However, when all of the genes of both the *TbMSP-B* and *GPI-PLC* families were deleted in the same BSF cell line (B^ −/−^, PLC ^−/−^), these cells were incapable of dividing in the differentiation medium, they had an altered morphology compared to either wild-type (WT) or single gene family-deleted cells (B ^−/−^ and PLC ^−/−^), and they exhibited a synergistic increase in VSG levels 96 h after differentiation initiation. Therefore, both TbMSP-B and GPI-PLC function in removal of VSG from the cell surface during differentiation of T. brucei BSF cells to the PRO form.

## Results

### Membrane Association and Surface Labeling of TbMSP-B

*Leishmania* MSP is GPI-anchored on the extracellular *Leishmania* promastigote surface, where it participates in several functions associated with *Leishmania* survival in its host [8,9]. To determine the corresponding cellular location of TbMSP-B, we first examined its amino acid sequence using the computer algorithm at http://mendel.imp.univie.ac.at/gpi/gpi_prediction.html, which predicted that nascent TbMSP-B has an N-terminal hydrophobic signal sequence (residues 1–30) and a C-terminal cleavage site for the addition of a GPI anchor at residue 530. The C-terminal sequence is positioned in front of a stretch of seven amino acids that constitute a hydrophilic spacer region, followed by a C-terminal hydrophobic region of 15 residues [12]. The presence of these N- and C-terminal hydrophobic sequences stimulated additional experiments to determine if TbMSP-B is membrane-associated and possibly located on the cell surface.

**Figure 1:**
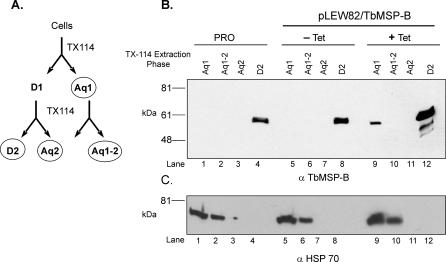
TbMSP-B Is an Integral Membrane Protein (A) Summary of the TX-114 extraction protocol used to separate trypanosome cytosolic proteins (Aq phases) and integral membrane proteins (D phases). Circled fractions were used in the western blots shown in the other panels. (B and C) PRO cells (lanes 1–4) and PRO cells containing integrated pLEW82/TbMSP-B, either not induced (lanes 5–8) or induced (lanes 9–12) with 1 μg tetracycline/ml, were subjected to TX-114 extraction and analyzed by western blots probed with antisera directed against (B) TbMSP-B (α TbMSP-B) and (C) HSP-70 (α HSP-70) as a control to detect a cytosolic protein and confirm purity of extraction phases.

Triton (TX)-114 extraction experiments were used to see if TbMSP-B fractionates as an integral membrane protein, as expected from its predicted GPI anchor. Figure 1A shows a schematic representation of the TX-114 extraction protocol used to separate the hydrophilic (aqueous phases) proteins from the integral membrane proteins (detergent phase). Briefly, PRO cells were solubilized in TX-114 and separated into an aqueous and detergent phase by raising the temperature above the TX-114 cloud point followed by centrifugation (Aq1 and D1 in Figure 1A). To ensure complete separation, these phases were again extracted with TX-114 and additional aqueous and detergent phases were isolated (Aq1–2, D2, and Aq2). The circled phases were then evaluated using western blots.

To facilitate these experiments and enhance the TbMSP-B signal, a PRO cell line was generated that contains the integrated plasmid, pLEW82/TbMSP-B, from which TbMSP-B is overexpressed when induced with tetracycline (see Materials and Methods). PRO cells without the integrated plasmid and PRO cells bearing the integrated pLEW82/TbMSP-B expression plasmid were grown in the absence (− Tet) or presence (+ Tet) of tetracycline and extracted with TX-114, and the aqueous and detergent phases were analyzed for the presence of TbMSP-B and HSP-70. Endogenous TbMSP-B was found exclusively in the detergent fractions of PRO cells and the uninduced overexpression PRO cell line (Figure 1B, lanes 4 and 8 respectively), indicating it is a membrane protein. When the pLEW82/TbMSP-B cell line was tetracycline-induced to overexpress the protein, TbMSP-B was found predominantly in the detergent phase. Although a small percentage was present in the intermediate Aq1 fraction, it did not appear in the fully extracted Aq1–2 fraction, indicating it is not a soluble cytosolic or peripheral membrane protein (Figure 1B, lanes 9–12). The smaller additional bands observed in lane 12 are likely degradation products of the highly synthesized protein. To verify the expected partitioning of cytosolic and membrane proteins in the procedure, the same phases were probed with antiserum directed against heat shock protein 70 (HSP-70) (Figure 1C). The presence of HSP-70 in the Aq1 and Aq1–2 phases shows that cytosolic proteins were separated from membrane proteins in the experiment and that TbMSP-B is a membrane protein.

**Figure 2:**
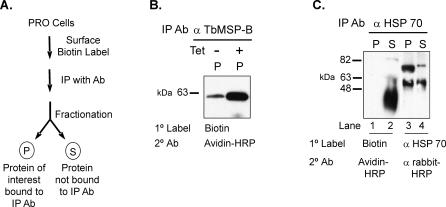
TbMSP-B Can Be Surface Biotinylated (A) Summary of the biotinylation and immunoprecipitation protocol used to detect proteins on the surface of PRO cells that are labeled with biotin. PRO cells were surface biotin-labeled with EZ-Link Sulfo-NHS-Biotin (Pierce), followed by cell lysis and immunoprecipitation (IP) of cell extracts with antiserum directed against TbMSP-B or HSP-70. (B) PRO cells bearing integrated pLEW82/TbMSP-B were not induced (− Tet) or induced (+ Tet) with 1 μg tetracycline/ml for 24 h prior to surface biotinylation and immunoprecipitation with affinity-purified MSP-B antiserum (α TbMSP-B). Proteins in the pellet fraction (P) were separated on an SDS-PAGE gel, and surface biotin-labeled proteins were visualized using an avidin-HRP secondary antibody. (C) PRO cells were surface biotinylated and rabbit HSP-70 antiserum (α HSP-70) was used to immunoprecipitate HSP-70 protein from the cell extracts. In lanes 1 and 2, the pellet (P) and supernatant (S) fractions are shown in which biotin was the primary label, followed by visualization of the biotin-labeled proteins with secondary avidin-HRP. The major band in lane 2 is biotin-labeled procyclin. Lanes 3 and 4 show a western blot in which the P and S fractions were incubated with rabbit HSP-70 antiserum followed by an anti-rabbit-HRP secondary antibody.

After multiple inconclusive attempts to determine the location of TbMSP-B using immunofluorescence microscopy, likely due to inability of our antisera to recognize the native protein during microscopy, we adopted the biochemical approach of utilizing biotin surface labeling to see if TbMSP-B is present on the cell surface. The general strategy for the biotin labeling/immunoprecipitation experiment is shown in Figure 2A. PRO cells were biotin-labeled, followed by immunoprecipitation with antiserum directed against the protein of interest and separation of the immunoprecipitated protein (pellet [P]) from the unbound proteins (supernatant [S]). PRO cells bearing stably integrated pLEW82/TbMSP-B were biotin-labeled after the culture had been split and grown either in the absence or presence of 1 μg tetracycline/ml medium for 24 h. The biotin-labeled cell extracts were immunoprecipitated with affinity-purified TbMSP-B antiserum and separated into pellet and supernatant fractions. Western blot analysis using a streptavidin-HRP antiserum to detect biotin-labeled proteins revealed that TbMSP-B in uninduced cells, i.e., likely the native TbMSP-B protein, was biotin-labeled during the procedure (Figure 2B). It also showed that the additional TbMSP-B expressed in the presence of tetracycline was likewise accessible to biotin labeling (Figure 2B, + Tet). To control for the possibility that all cellular proteins were biotin-labeled, and not only the surface proteins, PRO cells were biotin-labeled as before and cell extracts were immunoprecipitated with HSP-70 antiserum (Figure 2C) and BiP antiserum (not shown). Figure 2C, lanes 1 and 2, show the pellet (P) and supernatant (S) fractions from the biotin labeling/immunoprecipitation procedure in which the western blot was incubated with the streptavidin-HRP antibody. No biotin-labeled HSP-70 protein migrating at 70 kDa (and no BiP; not shown) was detected in either the P or S fractions, confirming that cytosolic proteins were not biotin labeled. The smeared band of protein below 48 kDa in lane 2 is likely the abundant biotin-labeled EP-procyclin that did not bind to the HSP-70 antiserum. The other weak band at 63 kDa could be endogenous TbMSP-B, while the distinct, weak band at 82 kDa represents an unidentified biotin-labeled protein. The presence of HSP-70 in each fraction was verified by performing a western blot of the fractions (Figure 2C, lanes 3 and 4). HSP-70 protein was identified in the pellet fraction (P) with an approximate size of 70 kDa along with a strong band of IgG molecules (55 kDa) carried through during the immunoprecipitation process. Thus, both endogenous and overexpressed TbMSP-B is accessible to surface biotinylation of live PRO cells.

### TbMSP-B Is a Zinc Metalloprotease

**Figure 3:**
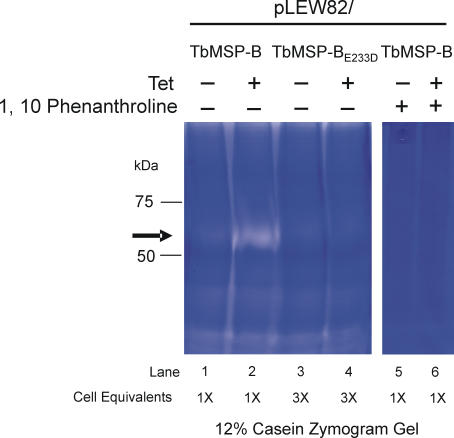
TbMSP-B Is a Zinc Metalloprotease (A) PRO cells containing integrated pLEW82/TbMSP-B or pLEW82/TbMSP-B_E233D_ were either not induced (− Tet) or induced (+ Tet) with 1 μg tetracycline/ml for 24 h, collected, and resuspended in non-reducing, denaturing Laemmli sample buffer. About 10^7^ cell equivalents were electrophoresed on a 12% casein zymogram gel followed by overnight incubation in development buffer in the absence (−) or presence (+) of the zinc metalloprotease inhibitor 1, 10 phenanthroline. After staining with Commassie blue dye and destaining, proteolytically cleaved gel substrate appeared as a clear band in the 60-kDa range (arrow and lane 2). The proteolytic activity was not observed in extracts from cells bearing pLEW82/TbMSP-B_E233D_ (lane 4). Note that three times the cell equivalents were loaded in assessing the proteolytic activity of this mutant TbMSP (lanes 3–4). Incubation of the zymogram gel in development buffer supplemented with 1, 10 phenanthroline resulted in severely diminished TbMSP-B protease activity (compare lanes 2 and 6).

TbMSP-B is predicted on the basis of its amino acid sequence to be a modified member of the metzincin class of zinc proteinases [13]. It has a conserved zinc metalloprotease catalytic site sequence of HEXXHXXG as well as other conserved residues, indicating that TbMSP-B could function as a protease in T. brucei. Endogenous TbMSP-B protease activity was initially difficult to detect, possibly because of low abundance of the protein. Thus, we used PRO cells bearing pLEW82/TbMSP-B to examine whether tetracycline-induced TbMSP-B has protease activity. The PRO cells were grown in the absence or presence of tetracycline for 24 h and processed for zymogram gel analysis. In the extracts of PRO cells overexpressing TbMSP-B, a clear proteolytic band appears at 60 kDa (Figure 3, lane 2, and arrow). When these same extracts were treated with 1, 10 phenanthroline, a zinc metalloprotease inhibitor, no proteolytic cleavage of the casein substrate in the 60-kDa region of the gel was observed (Figure 3, lanes 5 and 6). It should be noted also that TbMSP-B appears to have some substrate specificity because control *Leishmania* extracts exhibited MSP activity on both casein and gelatin substrates, whereas TbMSP-B only showed activity on a casein substrate (unpublished data). The significance of this observation is still under investigation.

*Leishmania* MSP metalloprotease activity depends on the presence of the HEXXH catalytic motif. McGwire and Chang [14] generated several mutations within the *Leishmania* MSP active site and determined that glutamate at position 265 was necessary for protease activity, but not for protein stability. To better define the TbMSP-B catalytic domain, we conducted a similar study by introducing a site-specific mutation within the predicted active site. Plasmid pLEW82/TbMSP-B was used as a template in the Stratagene QuickChange Site-Directed Mutagenesis procedure to generate a pLEW82/TbMSP-B_E233D_ mutant in which E in the catalytic motif was converted to D. The entire TbMSP-B coding region in this mutant was sequenced to confirm that no other mutation had been introduced. This mutant plasmid was stably integrated into the genome of PRO cells, selecting for bleomycin resistance. This cell line was grown in the absence (Figure 3, lane 3) and presence (lane 4) of tetracycline for 24 h and processed for zymogram gel analysis. The overexpressed TbMSP-B_E233D_ did not exhibit detectable protease activity (lane 4 and arrow) despite the presence of three times the amount of cell extract compared to the WT TbMSP-B extracts in Figure 3A, lanes 1 and 2. Western blot analysis of tetracycline-induced cells bearing pLEW82/TbMSP-B_E233D_ verified TbMSP-B_E233D_ expression under these conditions (unpublished data). These experiments demonstrate that TbMSP-B has zinc metalloprotease activity.

### Deletion of the TbMSP-B and GPI-PLC Genes

Initially, multiple experiments with BSF cells were conducted using RNAi directed against TbMSP-B mRNA to see what the effect of diminishing the steady state level of TbMSP-B would be on growth of BSF cells and on their differentiation to PRO cells in culture. However, in repeated experiments using cloned BSF cells bearing tetracycline-inducible TbMSP-B RNAi plasmids, it was not possible to completely eliminate TbMSP-B mRNA from the cells, especially during differentiation from BSF to PRO in culture. Thus, we decided to delete all of the TbMSP-B genes from the diploid genome of BSF cells, reasoning that BSF cells might not need TbMSP-B because preliminary western blots showed that in BSF cells it is in very low abundance or non-existent.

**Figure 4:**
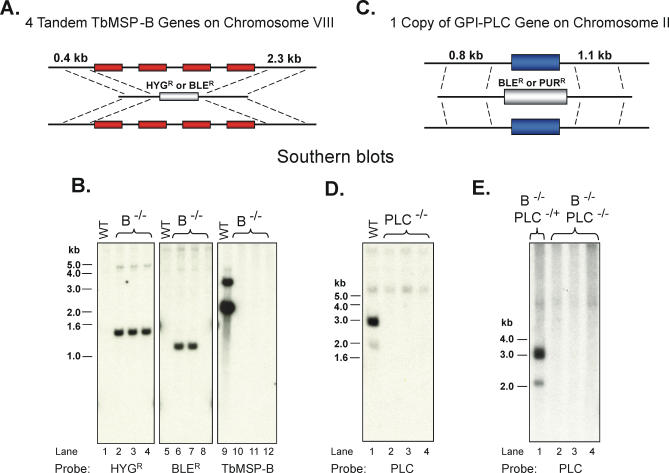
Generation of T. brucei BSF Cell Lines Bearing Gene Deletions (A) The strategy for deleting the cluster of four tandem TbMSP-B genes from both alleles of chromosome VIII utilized 0.4 kb of sequence upstream of the cluster and 2.3 kb of sequence downstream. The cassette used for the deletions contained either the HYG^R^ or BLE^R^ gene for selection with the appropriate antibiotic. (B) Analysis of three cloned MSP-B ^−/−^ cell lines (B ^−/−^). The HYG^R^ cassette was transfected first into the WT BSF cell line, followed by transfection with the BLE^R^ cassette. Southern blots of genomic DNAs from WT and three cloned B ^−/−^ cell lines cleaved with XhoI and StuI were probed with the coding regions of HYG^R^ (lanes 1–4) or BLE^R^ (lanes 5–8), and the same genomic DNAs cleaved with HindIII were probed with the coding sequence of TbMSP-B (lanes 9–12). Lanes 4, 8, and 12 show a cell line that does not contain the BLE^R^ gene but does lack all copies of the TbMSP-B gene (see text). This B ^−/−^ cell line was used in differentiation experiments and to generate doubly deleted B ^−/−^ PLC ^−/−^ cell lines (see [E]). (C) Strategy for generating PLC^−/−^ cell lines. Both alleles of the single-copy GPI-PLC gene were replaced with cassettes containing either the BLE^R^ or PUR^R^ gene flanked by a 0.8-kb upstream and 1.1-kb downstream genomic sequence. (D) Southern blot of genomic DNAs from WT BSF cells and three BSF PLC ^−/−^ cell lines cleaved with NdeI and probed with the PLC coding sequence. (E) Southern blots of genomic DNA from a B ^−/−^ cell line transfected with the GPI-PLC deletion cassettes. The B ^−/−^ cell line shown in lane 12 of (B) was transfected first with the PLC/BLE^R^ cassette (lane 1). The resulting B ^−/−^ PLC ^−/+^ cell line in lane 1 was then transfected with the PLC/PUR^R^ cassette to generate a doubly deleted cell line, three clones of which are shown in lanes 2–4. The genomic DNAs were cleaved with NdeI and probed with the PLC coding sequence.

The TbMSP-B gene (*TbMSP-B*) cluster is on chromosome VIII [10]. The four tandem *TbMSP-B*s in this cluster have 99% nucleotide identity and encode proteins with 99% amino acid identity. To delete the four tandem *TbMSP-B* genes on each chromosome VIII (Figure 4A), fragments of approximately 0.4 kb upstream of the gene cluster and 2.3 kb downstream were PCR-amplified from the genome, and cloned into sites on a plasmid that flank a DNA segment bearing either the hygromycin (*HYG* ^R^) or bleomycin (*BLE* ^R^) antibiotic resistance gene containing the 5′ and 3′ UTRs of the T. brucei actin gene. The resultant cassettes were excised from the plasmid and targeted sequentially into the *TbMSP-B* loci of BSF cells via electroporation. The expectation was that the first transfection (*HYG* ^R^ selection) would delete one allele and the second transfection (*BLE* ^R^ selection) would delete the other allele. HindIII-digested genomic DNAs from WT BSF cells, two independent BSF cell lines transfected sequentially with both *TbMSP-B* deletion cassettes, as well as one BSF cell line transfected only with the *HYG* ^R^ deletion cassette, were analyzed by Southern blot for the presence of *HYG* ^R^, *BLE* ^R^, and *TbMSP-B* coding sequence (Figure 4B). As expected, *HYG* ^R^ (∼1.5 kb) was present in the transfected cell lines (lanes 2–4), and absent from the WT BSF cell line (lane 1). When probed with *BLE* ^R^, two of the transfected cell lines were found to have this sequence (lanes 6 and 7), while the third cell line only transfected with the *HYG* ^R^ cassette did not contain it (lane 8). This cell line must have undergone two separate recombination events in which both alleles of the *TbMSP-B* gene cluster were replaced with the *HYG^R^* cassette. When DNAs from each of the cell lines were probed with *TbMSP-B*, WT cells showed strong hybridization to two *TbMSP-B* fragments of 2.1 and 3.2 kb as expected (lane 9), and *TbMSP-B* was absent from the other three cell lines (lanes 10–12). The *TbMSP-B* ^−/−^ cell line (B ^−/−^ cells) represented in lanes 4, 8, and 12 was used in future experiments and in generating cell lines in which the genes for both TbMSP-B and GPI-PLC were deleted (B ^−/−^ PLC ^−/−^ cells; Figure 4E and below).

GPI-PLC has been implicated in removing the VSG from the cell surface via cleavage of the GPI anchor during T. brucei differentiation [6,12,15]. This earlier work deleted the two allelic (single-copy) GPI-PLC genes from another parental cell line and showed that GPI-PLC is not essential for normal differentiation because this BSF PLC ^−/−^ cell line was still capable of differentiating and completing its life cycle. Figure 4C is a schematic representation of the GPI-PLC deletion strategy used in this work to delete both gene copies from the diploid T. brucei genome. The genes were replaced with cassettes containing either the puromycin (*PUR* ^R^) or the bleomycin (*BLE* ^R^) antibiotic resistance gene. The *PUR* ^R^ deletion plasmid was transfected first into WT BSF cells (Figure 4D, lane 1), followed by an additional transfection with the *BLE* ^R^ construct. Three GPI-PLC ^−/−^ cell lines were selected from this second transfection (lanes 2–4). Finally, to define the roles of both TbMSP-B and GPI-PLC during differentiation, a doubly deleted cell line (B ^−/−^ PLC ^−/−^), was generated using the B ^−/−^ cell line in which the *HYG* ^R^ cassette had replaced both alleles of the four-repeat *TbMSP-B* gene cluster (Figure 4B, lanes 4, 8, and 12). The B ^−/−^ cell line was first stably transfected with the linearized *PUR* ^R^ plasmid (Figure 4E, lane 1) followed by a second transfection with the linearized *BLE* ^R^ construct (Figure 4E, lanes 2–4). The establishment of the three different deletion cell lines, B ^−/−^, PLC ^−/−^, and B ^−/−^ PLC ^−/−^, allowed us to determine if these gene products, either alone or jointly, contribute to the release of VSG during differentiation and if either or both are necessary for differentiation.

### Growth and Differentiation of Deletion Cell Lines

**Figure 5:**
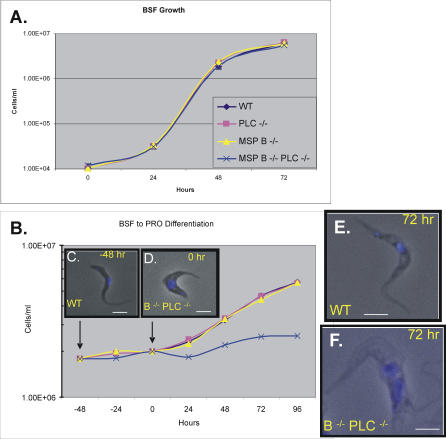
Single and Double Knock-Out BSF Cell Lines Grow Normally as BSF Cells, but B ^−/−^ PLC ^−/−^ Cells Exhibit a Growth Defect in Differentiation Medium (A) BSF WT (diamonds), B ^−/−^ (triangles), PLC ^−/−^ (squares), and B ^−/−^ PLC ^−/−^ (x) cells all grow at the same rate in bloodstream culture. (B) The doubly deleted B ^−/−^ PLC ^−/−^ cells (x) do not divide in the differentiation medium, whereas the WT, B ^−/−^, and PLC ^−/−^ cells have a doubling time of approximately 48 h. (C) Phase contrast imaging and DAPI staining of a WT cell prior to treatment with pCPTcAMP. (D) Phase contrast imaging and DAPI staining of a short, stumpy B ^−/−^ PLC ^−/−^ cell after 48 h of treatment with pCPTcAMP. (E) Phase contrast imaging and DAPI staining of a WT cell after 72 h in differentiation medium. (F) Phase contrast imaging and DAPI staining of a B ^−/−^ PLC ^−/−^ cell after 72 h in differentiation medium. The scale bar in (C–F) indicates 5 μm.

The growth of the WT BSF cells was compared to that of each type of the genomic deletion cell lines under normal BSF culture conditions over a 3-d period (Figure 5A). No significant difference in growth was observed for any cell line, confirming previous reports that TbMSP-B and GPI-PLC are not necessary for normal BSF cell growth [9,16], as well as establishing that BSF cells lacking both TbMSP-B and GPI-PLC grow normally under these conditions.

To test whether TbMSP-B or GPI-PLC or both are important during the differentiation process, all three deletion cell lines, as well as WT cells, were induced to differentiate from BSF to PRO form. The BSF cell lines were first treated for 48 h with a membrane-permeable derivative of cyclic adenosine monophosphate (cAMP), 8-(4-chlorophenylthio)-cAMP (dCPTcAMP), to stimulate conversion from proliferating long, slender BSF to non-proliferative, short, stumpy BSF [17], and then placed in PRO medium at 26 °C for differentiation from short, stumpy BSF to PRO form. Cell growth was monitored at 24-h intervals. No differences in growth rate was observed for the WT, B ^−/−^, and PLC ^−/−^ cell lines during differentiation, but the B ^−/−^ PLC ^−/−^ cell line was not able to divide at the same rate in the differentiation medium (Figure 5B, 0–96 h). During the first 48 h of differentiation (0–48 h) the three other cell lines multiplied with a doubling time of approximately 48 h, whereas the B ^−/−^ PLC ^−/−^ cell line showed a only modest increase in cell count to 2.6 × 10^6^ cells/ml (Figure 5B, 96 h) from the starting concentration of 2.0 × 10^6^ cells/ml (0 h). Cell morphology during differentiation was also documented for each cell line. All four cell lines had very similar morphology as long, slender BSF cells in the BSF growth medium (Figure 5C, WT BSF cells shown at time −48 h for reference). After 48 h growth in cAMP, long, slender cells had transformed to the short, stumpy form in all cell lines (Figure 5D, B ^−/−^ PLC ^−/−^ BSF cells shown for reference), but the doubly deleted cell line exhibited an altered morphology from 48 h through 96 h (Figure 5F versus Figure 5E). The B ^−/−^ PLC ^−/−^ cells were enlarged with multiple nuclei and kinetoplasts, and often had detached flagellae. The morphological differences of these cells compared to WT and the single-deleted cell lines, along with their lack of cell division, indicate that the presence of both TbMSP-B and GPI-PLC is essential for normal cell differentiation.

### TbMSP-B Expression and VSG Release during Differentiation

**Figure 6:**
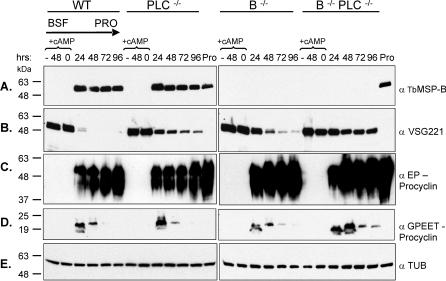
Expression of TbMSP-B, VSG, and Procyclin Proteins by WT and Gene-Deleted Cells during Differentiation from BSF to PRO Each cell line was treated with pCPTcAMP (cAMP) for 48 h (−48 to 0 h) to stimulate the transition of long, slender BSF cells to the short, stumpy form. At time 0, the short, stumpy BSF cells were centrifuged from BSF medium, resuspended in PRO medium containing 3 mM *cis*-aconitate/citrate, and placed at 26 °C to induce differentiation to PRO cells. Cells were collected at the time points indicated (h) and whole cell extracts were analyzed by western blots. The extracts were probed with antiserum directed against (A) TbMSP-B (α TbMSP-B), (B) VSG221 (α VSG221), (C) EP-procyclin (α EP-Procyclin), (D) GPEET-procyclin (α GPEET-Procyclin), and (E) α-tubulin (α TUB) as a control for equal loading. The lanes labeled PRO contain an extract of PRO cells grown continuously at 26 °C in PRO medium.

Many changes in gene expression are known to occur during T. brucei differentiation from BSF to PRO [18]. Among these changes, VSG transcription is substantially reduced during the first 4 h, and VSG protein is not detected in cells after approximately 48 h ([12,19] and Figure 6B). The two forms of procyclin, GPEET- and EP-procyclin, are expressed early in differentiation and replace the VSG as the predominant cell surface proteins. GPEET-procyclin then decreases, whereas the EP form continues to be expressed and occurs on the surface of PRO cells [20]. We have previously shown that TbMSP-B mRNA is present in both BSF and PRO cells [10]. Thus, we examined whether TbMSP-B protein is also found in both life cycle stages and what, if anything, happens to its expression during differentiation. Extracts from each of the four cell lines shown in the differentiation growth analysis in Figure 5B were analyzed by western blots for the presence of TbMSP-B (Figure 6A). WT BSF cells—both long, slender (−48 h) and short, stumpy forms (0 h)—expressed little, if any, detectable amounts of TbMSP-B protein despite the presence of its RNA ([10] and unpublished data), but TbMSP-B was readily detected 24 h after the cells were placed in PRO medium. TbMSP-B protein expression was highest at the 24-h time point, but remained higher for the entire 96-h differentiation period than in PRO cells continually grown at 26 °C in PRO medium. PLC ^−/−^ cells showed a similar pattern of TbMSP-B expression. No TbMSP-B was detected at any time point in either the B ^−/−^ or the B ^−/−^ PLC ^−/−^ cell lines.

**Figure 7:**
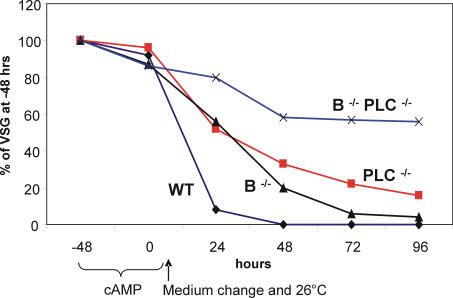
VSG Levels during BSF-to-PRO Differentiation The VSG bands on the western blot shown in Figure 6B were quantitated by densitometry for each of the four cell lines during BSF-to-PRO differentiation. The VSG intensities were normalized to the tubulin levels at each time point and the initial VSG level (−48 h) for each cell line was set at 100%. The VSG level shown for each time point is a percentage of initial VSG levels. The amounts of VSG remaining in B ^−/−^ (triangles), PLC ^−/−^ (squares), and B ^−/−^ PLC ^−/−^ (x) cells all decline more slowly than that of WT (diamonds) cells during differentiation. At 96 h, B ^−/−^ PLC ^−/−^ cells have approximately 60% VSG remaining, compared to 16%, 5%, and 0% in PLC ^−/−^, B ^−/−^, and WT cells, respectively.

VSG221, the VSG expressed by these BSF cells, was gone from WT cells by 48 h (Figure 6B) but remained in PLC ^−/−^ and B ^−/−^ cells through the 96 h-time point at gradually decreasing levels. The reason for the presence of two VSG221 protein bands in WT and B ^−/−^ cell extracts (Figure 6B) is not known. Treatments with two separate reducing compounds, beta-mercaptoethanol and dithiothreitol, did not eliminate the double bands. When both *TbMSP-B* and *PLC* were deleted from the genome, about 60% of the VSG remained on the cells 96 h after differentiation was initiated (Figures 6B and 7). Thus, both TbMSP-B and GPI-PLC participate in the loss of VSG during differentiation and may act synergistically. Figure 7 summarizes densitometry measurements of the loss of VSG from WT cells and the three deletion cell lines. Note that the B ^−/−^ PLC ^−/−^ cells retain about 60% of their VSG after 48 h in the differentiation medium, and do not lose any more during the next 48 h. During this same time, these cells acquire abnormal shapes and do not divide (Figure 5B and 5F).

The procyclin proteins were expressed as expected in the differentiating cells. EP-procyclin was detected in all cell lines within 24 h of transfer to PRO medium at 26 °C (Figure 6C). However, more EP-procyclin was present in both of the B ^−/−^ cell lines than in the WT cells throughout the differentiation process. The significance of this observation is still under investigation, but it might suggest that TbMSP-B cleaves procyclin to a small extent. GPEET-procyclin expression occurred early in differentiation as previously reported [20] and disappeared in WT and PLC ^−/−^ cells by 72 h (Figure 6D). This protein expression pattern was also slightly modified in both of the B ^−/−^ cell lines in that more GPEET-procyclin was present at the later time points. Figure 6E shows a western blot probed with tubulin antiserum to confirm equivalent loading of cell samples.

### The Differentiation Medium Contains VSG Protein

**Figure 8:**
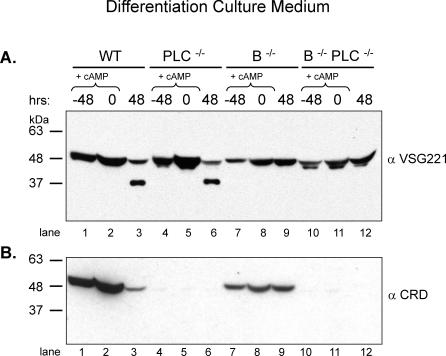
Released VSG221 Protein Occurs in the Culture Medium of BSF Cells and BSF-to-PRO Differentiating Cells Cultured logarithmic WT and gene-deleted BSF cells were subjected to centrifugation and supernatants were collected prior to pCPTcAMP (cAMP) addition (−48), prior to resuspension of cells in PRO medium (0) and 48 h after differentiation was initiated. Aliquots of the supernatants were probed in western blots with (A) antiserum against VSG221 (α VSG221) and (B) then stripped and reprobed with antiserum against the cross-reactive determinant (α CRD) found on GPI-anchored proteins hydrolyzed by GPI-PLC.

Previous studies of a transgenic PRO cell line engineered to express a recombinant VSG revealed that RNAi knock-down of TbMSP-B mRNA resulted in reduced release of the VSG from the cell surface into the medium [10]. This information, along with the fact that TbMSP-B only appears when BSF cells begin to differentiate, led us to examine the VSG present in the culture medium to determine whether TbMSP-B is responsible for release of a specific form of VSG during differentiation. Three time points were selected: −48 h (long, slender BSF cells), 0 h (short, stumpy BSF cells), and 48 h after the start of differentiation. Western blot analysis of the culture medium from WT cells probed with VSG221 antiserum showed a 48-kDa VSG band at the −48 and 0 h time points (Figure 8A, lanes 1 and 2), and two VSG bands (48- and 37-kDa) after 48 h of differentiation (lane 3). In the absence of GPI-PLC, a similar pattern of 48- and 37-kDa VSG bands is seen (Figure 8A, lanes 4–6). However, in the absence of TbMSP-B (B ^−/−^ cells and B ^−/−^ PLC ^−/−^ cells), the 37-kDa VSG fragment does not occur in the differentiation medium (Figure 8A, lanes 7–9 and 10–12), suggesting TbMSP-B is responsible for it. The VSG in the medium of the B ^−/−^ PLC ^−/−^ cell line (lanes 10–12) occurs as a double band in the 48-kDa region, for reasons as yet unknown. The relative intensities of the bands in the three lanes for each cell line (the −48, 0, and 48 lanes) varied from one experiment to another (not shown), so it was not possible to ascribe significance to these relative VSG intensities in the medium. In contrast, the relative intensities of the VSG bands derived from the differentiating cells at different time points (Figure 6B) varied little among independent experiments.

The filter shown in Figure 8A was stripped and reprobed with antiserum directed against the cross-reacting determinant (CRD) found on VSG protein that has been cleaved by GPI-PLC [21]. This analysis was conducted to confirm that the 37-kDa fragment is not due to release by a phospholipase. Figure 8B shows that the CRD antiserum detected the 48-kDa VSG from the WT and B ^−/−^ cells but did not recognize the 37-kDa VSG fragment, again suggesting this smaller VSG fragment occurs as a result of proteolytic release. The CRD antiserum did not detect proteins in the medium samples of either of the PLC ^−/−^ cell lines (Figure 8B, lanes 4–6 and 10–12) even though VSG was present in those samples. This result suggests that additional enzymes may also be involved in the release of VSG into the medium.

### TbMSP-B Complementation Restores VSG Release

**Figure 9:**
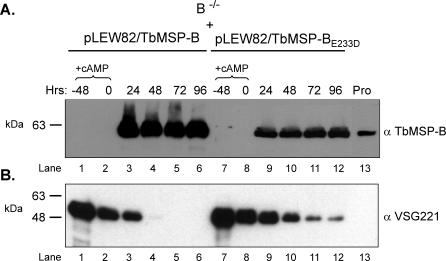
Complementation of B ^−/−^ Cells with TbMSP-B Encoded on an Integrated Plasmid BSF B ^−/−^ cells were stably transfected with linearized pLEW82/TbMSP-B or pLEW82/TbMSP-B_E233D_ expression plasmid and stimulated to differentiate as previously described except that 1 μg tetracycline/ml was added at times 0 and 48 h to induce TbMSP-B or TbMSP-B_E233D_ expression from the integrated plasmid. Extracts prepared from cells collected at the indicated times were probed in western blots with antiserum against (A) TbMSP-B (α TbMSP-B) and (B) VSG221 (α VSG221). The lane labeled PRO contains an extract of PRO cells grown continuously at 26 °C in PRO medium.

To confirm that TbMSP-B contributes to the release of VSG during differentiation, the WT or mutant *TbMSP-B* gene was added back to the genome of the B ^−/−^ cell line (Figure 9). Plasmids pLEW82/TbMSP-B or pLEW82/TbMSP-B_E233D_ were stably transfected into the rRNA gene locus of B ^−/−^ cells to generate B ^−/−^ (*TbMSP-B*) or B ^−/−^ (*TbMSP-B*_E233D_) cells. These two cell lines were taken through the same differentiation process as described above. Cell samples were collected at 24-h intervals and analyzed for TbMSP-B and VSG protein by western blot. Tetracycline was added to each differentiating cell culture at 0 and 48 h to induce the expression of TbMSP-B or TbMSP-B_E233D_ from the integrated pLEW82 plasmid. Figure 9A shows that TbMSP-B and TbMSP-B_E233D_ were expressed from 24 h on through the differentiation. TbMSP-B_E233D_ was expressed at slightly lower levels than WT TbMSP-B, a repeatable observation, but was still expressed at levels higher than endogenous TbMSP-B in PRO cells (Figure 9A, lane 13). The complementation of the B ^−/−^ cell line with WT TbMSP-B resulted in the loss of VSG by 48 h (Figure 9B, lanes 1–6), similar to that observed when WT BSF cells differentiate. In contrast, addition of TbMSP-B_E233D_ did not lead to a similar VSG loss as the protein was detected through 96 h (Figure 9B, lanes 7–12), similar to that of B ^−/−^ cells.

**Figure 10:**
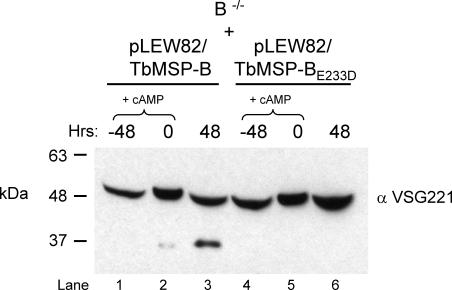
Truncated VSG221 Protein Occurs in the Culture Medium of Differentiating B ^−/−^ Cells When Complemented with TbMSP-B (Lanes 1–3) but Not When Complemented with TbMSP-B_E233D_ (Lanes 4–6) Aliquots of the culture medium were collected as previously described and probed in a western blot with antiserum directed against VSG221 (α VSG221).

In addition to the above evidence, western blot analysis confirmed TbMSP-B as necessary for release of the 37-kDa VSG fragment into the medium when WT BSF cells differentiate to the PRO form, as observed in Figure 8A, lanes 3 and 6. Complementation of B ^−/−^ cells with the WT TbMSP-B (Figure 10, lanes 1–3) again resulted in appearance of a 48-kDa VSG protein and a 37-kDa VSG fragment the medium. In these experiments, a small amount of the 37-kDa fragment was present prior to the addition of tetracycline at 0 h, when no TbMSP-B protein is detected. The simplest explanation of this result is that in the absence of tetracycline there is a low level of leaky *TbMSP-B* expression from integrated pLEW82/TbMSP-B, which is below our detection capabilities with this TbMSP-B antibody. The 37-kDa VSG fragment is more abundant at 48 h and is only present when WT TbMSP-B was reintroduced. The 48-kDa VSG protein is the only form present in the culture medium from B ^−/−^ cells complemented with TbMSP-B_E233D_ (Figure 10, lanes 4–6). Thus, an active TbMSP-B protease is necessary for release of the 37-kDa VSG fragment during differentiation.

### Cysteine Protease Treatment of Differentiating Cells

**Figure 11:**
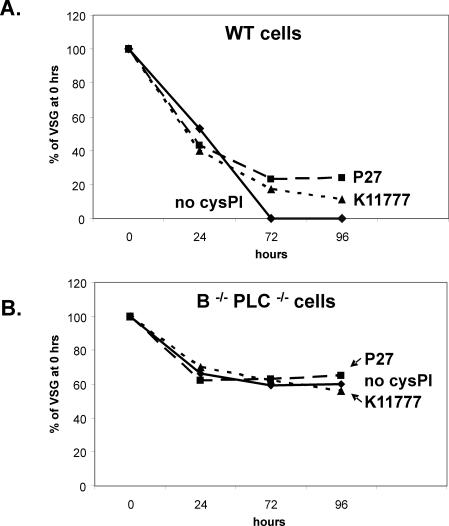
VSG Levels during BSF-to-PRO Differentiation in the Presence of Cysteine Protease Inhibitors WT (A) and B ^−/−^ PLC ^−/−^ (B) BSF cells were stimulated to differentiate to PRO cells as described except that 2 μM concentrations of the inhibitors P27 and K11777 were either added or not added (no cysPI) to the PRO differentiation medium at time 0. Cells were collected at the indicated times and extracts analyzed by western blots for VSG and tubulin, similar to the blots shown in Figure 6B and 6E. VSG levels were quantitated at each time point, the VSG intensities were normalized to the tubulin levels, and the initial VSG levels (0 h) were set at 100%, similar to that shown in Figure 7. The VSG level shown for each time point is a percentage of initial VSG level. In WT cells (A), P27 and K11777 interfere with the loss of about 20% and 10% of the VSG, respectively, at 96 h, whereas in B ^−/−^ PLC ^−/−^ cells (B) the cysteine protease inhibitors do not have a detectable effect.

The observation that about 40% of the VSG is still released from differentiating B ^−/−^ PLC ^−/−^ cells (Figure 7) prompted us to investigate whether cysteine protease inhibitors would interfere with this VSG release, because it has been reported [22] that cysteine protease inhibitors partially interfere with VSG release during differentiation. WT and B ^−/−^ PLC ^−/−^ BSF cells were induced to differentiate as described for Figure 6, except that the cysteine protease inhibitors P27 or K11777 were added to a final concentration of 2 μM [22] when the cells were transferred to PRO medium. As shown in Figure 11, western blot analysis of cell extracts during differentiation was used to determine the VSG221 levels in the absence of the cysteine protease inhibitors (no cysPI), or in the presence of P27 or K11777. In the absence of these inhibitors, VSG was completely released from WT cells by 72 h of differentiation (Figure 11A). The addition of each of the cysteine protease inhibitors increased the levels of VSG present on the cells at 96 h of differentiation from 0% to 10%–20%, consistent with, but not as dramatic as, the 50% seen previously [22]. However, treatment of B ^−/−^ PLC ^−/−^ cells with these inhibitors during differentiation (Figure 11B) did not reveal a significant difference in VSG levels compared to non-treatment (also compare with Figures 6B and 7).

Finally, western blot analysis (not shown) of the differentiation culture medium in the presence of the two cysteine protease inhibitors revealed an identical VSG pattern in the medium to that observed when examining WT and B ^−/−^ PLC ^−/−^ supernatants (Figure 8A). The inhibitor treatment of WT cells had no effect on the presence of both the 48-kDa and the 37-kDa VSG proteins in the medium 48 h after the start of differentiation. Likewise, the medium of B ^−/−^ PLC ^−/−^ cells treated with these inhibitors contained the same 48-kDa VSG as the untreated cells.

## Discussion

Here, we used a monomorphic BSF cell line deleted in the genes for either TbMSP-B or GPI-PLC, or both, to show that (i) TbMSP-B is the zinc metalloprotease involved in VSG loss during differentiation, and (i) TbMSP-B and GPI-PLC participate synergistically in VSG loss during BSF-to-PRO differentiation. The differential expression of TbMSP-B is consistent with its involvement in VSG loss during differentiation. It is not expressed to an appreciable extent in long, slender or short, stumpy BSF cells, but it appears a few hours after the start of differentiation and remains present in established PRO cells (Figure 6A). TbMSP-B is also localized on the cell surface in PRO cells as determined by biotin-labeling experiments (Figure 2B). Since we were unable to successfully use immunolocalization with the anti-TbMSP-B serum to determine the exact location of TbMSP-B, its site on the cell surface remains under investigation, but several possibilities exist. It could be located in the flagellar pocket during differentiation, as suggested by Ziegelbauer et al. [6], positioning it for access to the continually endocytosed and recycled VSG protein [23]. Alternatively, it might be distributed across the cell membrane during the early hours of differentiation when VSG is removed from the cell surface.

Glutamate at position 265 (E_265_) has been shown in a *Leishmania* MSP to be required for its protease activity, whereas changes at several other residues reduced protein stability but not enzyme activity [14]. We utilized a similar mutation strategy to examine the equivalent glutamate within the conserved HEXXH sequence (-H_232_EIAH_236_-) of TbMSP-B [10]. The conservative substitution of E_233_ to D_233_—an amino acid differing by only one methylene group—resulted in loss of protease activity (Figure 3A). Interestingly, TbMSP-B could cleave a casein substrate in the zymogram gel, but did not yield a proteolytic band in a gelatin zymogram gel, as does *Leishmania* MSP. This observation provides a starting point for assessing TbMSP-B's substrate and sequence specificity.

Initially our differentiation experiments involved the direct transfer of logarithmically growing BSF cells at 37 °C to PRO medium at 26 °C supplemented with 3 mM *cis*-aconitate and 3 mM citrate [24]. In these experiments, decreasing amounts of VSG were detected in WT cell extracts throughout the first 4 d of differentiation (not shown). We subsequently changed the differentiation protocol to first stimulate the transformation of logarithmic, long, slender BSF cells to the non-proliferating, short, stumpy form by adding a membrane-soluble cAMP [17] prior to transfer to differentiation medium. This alternative differentiation condition, which resulted in a more synchronized differentiation in which the VSG was fully released from the WT cells by 48 h, was used in all of the experiments described here.

### PLC ^−/−^ Cell Differentiation

Deletion of the GPI-PLC genes from the genome of BSF cells led to a delay in loss of VSG from differentiating cells (PLC ^−/−^ in Figures 6B and 7). This delayed loss differs somewhat from an earlier observation by Webb et al. [16], in which another PLC ^−/−^ cell line exhibited full release of VSG during differentiation. This disparity could be due to several factors, including differences in the two laboratory cell lines, which express different VSGs. The EATR 1125 trypanosome cell line utilized in [16] expressed a VSG(s) that cross reacted with antibodies against AnTat 1.1, 1.2, and 1.3, whereas the BSF cells used here express MITat 1.2 (VSG221). These VSG proteins are members of different VSG domain types, with AnTat 1.1 being a domain type 1 variant and MITat 1.2 a domain type 2 variant [6,25]. It has been proposed that removal of VSG during differentiation might use different mechanisms depending on the type of VSG present on the cell surface [7].

Examination of the culture medium of differentiating cells also demonstrates that GPI-PLC is responsible for the release of VSG into the medium during differentiation. The supernatants from both BSF PLC ^−/−^ and differentiating PLC ^−/−^ cells do not contain the VSG protein recognized by the anti-CRD antibody, the hallmark of PLC cleavage (Figure 8B), but the medium does contain full-length, or nearly full-length, VSG protein of approximately 48 kDa, suggesting there is an additional method of VSG release separate from GPI-PLC and TbMSP-B.

### TbMSP-B ^−/−^ Cell Differentiation

The absence of TbMSP-B in differentiating cells led to a delay in VSG loss, similar to that observed with PLC ^−/−^ cells (Figure 7), and correlated with the absence of the proteolytically cleaved VSG fragment in the culture medium (Figure 8). This 37-kDa VSG fragment did not react with anti-CRD antibody and therefore likely represents the N-terminal portion of the protein. Its absence from the TbMSP-B ^−/−^ cell lines and its reappearance in cells when a TbMSP-B gene was reintroduced into the genome (Figure 9) confirms TbMSP-B's role in generating the smaller VSG fragment. The exact site of its VSG221 cleavage remains to be determined. One cleavage constraint is that TbMSP-B and the VSG are both GPI-anchored to the membrane, so the catalytic site of TbMSP-B may have access to only certain segments of the VSG in vivo. It is also worth noting that the peptide substrate specificity of *Leishmania* MSP has been extensively studied and found to be relatively non-specific [26,27].

### TbMSP-B ^−/−^ PLC ^−/−^ Cell Differentiation

The slowed loss of VSG from each of the single gene knock-out cell lines was exacerbated when both TbMSP-B and GPI-PLC were absent from the cells. The VSG levels in extracts after 96 h of differentiation were not additive in this doubly deleted cell line; instead, TbMSP-B and GPI-PLC appear to act synergistically. At 96 h post-differentiation, the B ^−/−^ and PLC ^−/−^ cells have 5% and 16% of the VSG remaining, whereas the B ^−/−^ PLC ^−/−^ cells have 60% remaining (Figure 7). Currently there is no reason to expect that these two proteins interact in a cooperative manner during differentiation, but both can act to remove the VSG in a step essential for completion of the life cycle. When additional localization studies on TbMSP-B and GPI-PLC can be conducted, the proximity of these two proteins can be addressed.

Virtually no change occurs in the amount of VSG in extracts of B ^−/−^ PLC ^−/−^ cells between 48 and 96 h of differentiation (Figure 7); it remains at about 60%. During this time the cells have acquired a large amount of EP-procyclin, ceased to divide, and assumed an abnormal morphology (Figures 5B, 5F, and 6C). We have not examined these B ^−/−^ PLC ^−/−^ cells further to see whether, in addition to the acquisition of EP-procyclin, they have undergone the metabolic changes associated with differentiation to PRO cells. However, it is likely these cells cannot cope with the massive amount of EP-procyclin being made at a time when they are unable to eliminate 60% of their VSG to make room for the EP-procyclin on the surface. The result is that the cells shut down cell division, yet remain viable in the PRO medium for several days. Eventually, the cells die (not shown).

Another point is that even in the absence of TbMSP-B and GPI-PLC, about 40% of the VSG is lost by 48 h of differentiation (Figure 7). Some of this VSG loss is likely due to degradation and/or internalization [23], but some may also be due to other unknown cellular components that might contribute to VSG loss. Consistent with this possibility is the fact that the medium of B ^−/−^ PLC ^−/−^ cells contains full-length, or nearly full-length, VSG molecules of about 48 kDa that do not have the CRD (Figure 8), and thus are not the result of cleavages by TbMSP-B and GPI-PLC. We investigated whether cysteine proteases might contribute to this 40% loss, as was suggested by another report [22]. We confirmed that indeed inhibitors of cysteine proteases prevent loss of some VSG from differentiating WT cells either directly or indirectly (Figure 11A), but they did not interfere with that 40% loss of VSG from differentiating B ^−/−^ PLC ^−/−^ cells (Figure 11B). Again, it is worth noting that these experiments were conducted with a different BSF cell line expressing a different VSG than were the original cysteine protease inhibitor studies [22], and there may be VSG-dependent variability in the mechanisms of release. Thus, we conclude that TbMSP-B and GPI-PLC are clearly involved in the cellular strategy for removing VSG quickly during BSF-to-PRO differentiation, but the trypanosome may have additional specific mechanisms designed to achieve full VSG release in a timely manner [7].

## Materials and Methods

### Trypanosome strains and culture conditions.

The *T. brucei* BSF “single marker” (SM) trypanosome cell line (T7RNAPol TetR NEO) and the PRO 29–13 cell line (T7RNAPol NEO TetR HYG) were gifts from G. A. M. Cross (Rockefeller University, New York). Both of these cell lines constitutively express T7 RNA polymerase and the tetracycline repressor. BSF SM cells were maintained in HMI-9 medium [28] supplemented with 10% heat-inactivated fetal calf serum (HIFCS), and PRO 29–13 cells were maintained in SDM-79 medium [9] supplemented with 10% HIFCS.

The pLEW82 expression plasmid for T*. brucei* [29] was a gift from G. A. M. Cross. The TbMSP-B coding sequence was subcloned into pLEW82 at its unique HindIII and BamHI restriction sites. The resultant plasmid was linearized at its unique NotI site and stably integrated into the T. brucei rDNA spacer region for subsequent tetracycline-induced expression of TbMSP-B. For plasmid transfections into BSF cells, the cells were electroporated [30] with 10 μg NotI-linearized plasmid, and stable transfectants were selected in medium containing 5 μg G418/ml, 5 μg phleomycin/ml, 5 μg hygromycin/ml, and 0.5 μg puromycin/ml under conditions described previously [10]. PRO cells were also transfected with 10 μg NotI-linearized plasmid as described [31], and stable transfectants were selected in medium containing 15 μg G418/ml and 2.5 μg phleomycin/ml.

### Differentiation of bloodstream trypanosomes.

BSF cells were stimulated to transform from dividing long, slender cells to non-dividing short, stumpy cells by the addition of the membrane-permeable derivative of cAMP, 8-(4-chlorophenylthio)-cAMP (pCPTcAMP), to a final concentration of 0.4 mM in HMI-9 medium + 10% HIFCS media for 48 h [17]. After 48 h, the short, stumpy cells were centrifuged from the BSF medium and resuspended in SDM-79 medium + 10% HIFCS supplemented with 3 mM *cis*-acontitate/3 mM citrate to induce differentiation to the PRO form [24]. Cysteine protease inhibitors P27 (morpholinourea-phenylalanine homophenylalanine-benz-α-pyrone) and K11777 (*N*-methyl-piperazine-phenylalanine-homophenylalanine-vinylsulfone-phenyl) (gifts from James McKerrow, University of California at San Francisco) were added to SDM-79 supplemented with *cis*-aconitate and citrate at a concentration of 2 μM during differentiation for experiments described in Figure 10.

### Protein extracts, supernatant collection, and western blot analysis.

BSF and PRO cells were collected via centrifugation followed by a wash in 1x PBS. Total protein extracts were prepared by resuspending cell pellets in 1x Laemmli sample buffer and boiling for 5 min. Protein extracts were separated by SDS-PAGE and analyzed by western blot [32]. In addition to the antiserum directed against recombinant TbMSP-B, other antisera used in the western blots were directed against HSP-70 and BiP (both kind gifts from Jay Bangs, University of Wisconsin-Madison), VSG221 (the kind gift of George Cross), EP-procyclin and GPEET-procyclin (both kind gifts from Terry Pearson, University of Victoria), the CRD of the GPI anchor (the kind gift of Paul Englund, Johns Hopkins University), and α-tubulin (AB-1, Oncogene). Secondary antibodies linked to horseradish peroxidase were used to detect proteins via chemiluminescence. Media supernatants were collected from cell cultures as previously described [7]. Briefly, cell cultures were centrifuged for 2 min to pellet cells and the supernatant was added in a 10:1 ratio to a solution containing 1% NP40, 0.5% deoxycholate, and 0.1% SDS. A 100x protease inhibitor cocktail (Sigma) was added to 1x final concentration and samples frozen at −20 °C until used. Samples were then analyzed by western blot as described above.

### Preparation and affinity purification of anti-TbMSP-B antiserum.

The TbMSP-B coding sequence from nucleotides 157 through 1125 was PCR-amplified from plasmid pLEW82/TbMSP-B using primers designed with a BamHI site in the 5′ primer and an XhoI site in the 3′ primer. The PCR fragment was subcloned into pCR2.1-Topo (Invitrogen) and excised using BamHI and XhoI. The purified TbMSP-B coding sequence was then subcloned into purified BamHI/XhoI-digested pET32A plasmid (Novagen). The pET32A/TbMSP-B plasmid was transformed into E. coli DE3 cells (Novagen) and induced to overexpress the pET32A/TbMSP-B chimeric protein with 1 mM IPTG as per the Novagen pET System Manual. The pET32A/TbMSP-B fusion protein was insoluble in DE3 cells and localized to inclusion bodies. Inclusion bodies were isolated as described [33] and injected into a New Zealand White rabbit at 2 mg/injection, supplemented with Freud's Complete Adjuvant [34]. Two additional booster injections were made at 4 wk and 6 wk before complete collection of antisera.

Affinity purification of the anti-TbMSP-B antiserum was performed using HIS-purified pET32A/TbMSP-B fusion protein. The fusion protein was isolated using a Novagen HIS-Bind Kit. The HIS-purified protein was electrophoresed on an SDS-PAGE gel and transferred to a PVDF membrane. A strip of membrane corresponding to the size of pET32A/TbMSP-B fusion protein was cut from the membrane and used in the affinity purification. The PVDF strip with the bound fusion protein was incubated with the rabbit anti-TbMSP-B serum and washed first in 500 mM NaCl/50 mM Tris-HCl (pH 7.4), followed by washes in 100 ml NaCl/50 mM Tris-HCl (pH 7.4). Antibody bound to the membrane was removed with 50 mM glycine (pH 2.5) at 4 °C and brought back to pH 7.4 with 2 M Tris-HCl [35]. Affinity-purified antiserum (α-TbMSP-B) was stored at 4 °C for use.

### Zymogram gels.

PRO cells stably transfected with linearized pLEW82/TbMSP-B expression plasmid were cultured in the absence or presence of 1 μg tetracycline/ml to overexpress TbMSP-B for 24 h. Cell samples were collected and washed twice in 1x PBS. Whole cells were lysed in a non-reducing, denaturing sample buffer and passaged through a 27-gauge needle three times prior to running on a Bio-Rad 12% casein zymogram gel at 4 °C. The zymogram gel was subsequently washed twice in 2% TX-100, rinsed briefly in double-deionized H_2_O, and washed once in Bio-Rad Zymogram development buffer, followed by incubation in development buffer at 37 °C [36]. Staining with Commassie blue and destaining in 40% methanol/10% acetic acid revealed proteolytic bands.

### Construction of TbMSP-B and GPI-PLC gene-deletion plasmids.

The *TbMSP-B* and *GPI-PLC* gene-deletion plasmids were generated using a core plasmid construct containing the rRNA promoter, actin (*ACT*) 5′ UTR, and the bleomycin-resistance (*BLE*) gene from the RNA interference plasmid p2T7^ti^A/GFP [37]. The genes encoding hygromycin phosphotransferase (*HYG*), streptothricin acetyltransferase (*SAT*), and puromycin acetyltransferase (*PUR*) were used to replace the *BLE* gene at unique restriction sites introduced during cloning.

A 0.4-kb genomic sequence preceding the cluster of four tandem *TbMSP-B* genes on chromosome VIII was PCR-amplified with primers homologous to the sequences about 80 bp and 480 bp upstream of the start codon of the first *TbMSP-B* gene in the cluster. A 2.26-kb genomic sequence following the cluster was PCR-amplified with primers homologous to portions of the 3′ UTR and intergenic region about 550 bps to 2,810 bps downstream of the last *TbMSP-B* gene in the cluster. These upstream and downstream sequences were subcloned in the gene-deletion plasmid to direct recombination and removal of the entire *TbMSP-B* four-gene cluster. Genomic sequences flanking *GPI-PLC* were generated similarly to the *TbMSP-B* target sequences, but contained ∼0.84 kb upstream sequence and ∼1.14 kb downstream sequence designed to replace the single copy *GPI-PLC* on chromosome II.

### Southern blots.

Genomic DNAs isolated from BSF cells were digested with restriction enzymes, separated on a 1% agarose gel, depurinated in 0.25 M HCl, denatured in 0.4 M NaOH, transferred to nylon membrane (Hybond, Amersham International), and immobilized one time by UV irradiation. [^32^P]-radiolabeled DNA probe was added to a 50% formamide, 6X SSC, 10X Denhardt's solution, 0.2% SDS hybridization solution with 150 μg single-stranded salmon sperm DNA/ml at 50 °C overnight. Final washing conditions were in 0.1% SSC, 0.1% SDS at 60 °C.

### TX-114 extraction, biotin labeling, immunoprecipitation.

PRO cells were extracted with TX-114 as previously described [36] and aqueous and detergent phases were electrophoresed on SDS-PAGE in a 3:5 ratio with 2x SDS reducing sample buffer. Western blot analysis using the protocol described above was performed to visualize TbMSP-B and HSP-70 proteins in each phase. Biotin labeling (EZ-Link Sulfo-NHS-Biotin, Pierce) of PRO cells was conducted according to EZ-Link instructions and cells were lysed in 0.5% TX-100 for 30 min. The supernatant was then pre-cleared using rabbit pre-immune serum and immunoprecipitated with affinity-purified TbMSP-B antiserum, HSP-70, or BiP antisera [38]. Protein bound to the immunoprecipitating antiserum was released by boiling in 1x SDS Laemmli sample buffer. Biotin-labeled proteins were separated on a SDS-PAGE gel, blotted to nitrocellulose and the membrane incubated with a streptavidin-HRP antibody (Sigma). HSP-70 and BiP immunoprecipitations were also analyzed in western blots probed with antisera directed against HSP-70 and BiP.

## Supporting Information

### Accession Numbers

UniProt accession numbers (http://www.ebi.uniprot.org/) for the main T. brucei proteins utilized or mentioned in this research are α-tubulin (Q4GYY5); EP-procyclin (P08469); GPEET-procyclin (Q581F9); GPI-PLC (P09194); TbMSP-B (Q580G0); and VSG221 (P26332). GenBank (http://www.ncbi.nlm.nih.gov/Genbank/index.html) accession numbers for the genes deleted from the T. brucei genome in this research are *GPI-PLC* (XM_946646) and *TbMSP-B* (XM_841902, XM_841903, XM841904, XM841905).

## Abbreviations

BSF: bloodstream form
cAMP: cyclic adenosine monophosphate
CRD: cross-reacting determinant
GPI: glycosylphosphatidylinositol
GPI-PLC: glycosylphosphatidylinositol phospholipase C
HSP-70: heat shock protein 70
MSP: major surface protease
PRO: procyclic
RNAi: RNA interference
TbMSP-B: T. brucei major surface protease-B
TX-114: triton X-114
VSG: variant surface glycoprotein
WT: wild-type
